# Supplementary material for: FBXO24 ensures male fertility by preventing abnormal accumulation of membraneless granules in sperm flagella
Source: bioRxiv. 2023 Nov 10:2023.11.10.566635. Preprint. [Version 1] doi: 10.1101/2023.11.10.566635 (PMC10659433; doi:10.1101/2023.11.10.566635)
Supplement: Supplementary file 1 [file NIHPP2023.11.10.566635v1-supplement-1.pdf]

bioRxiv preprint doi: https://doi.org/10.1101/2023.11.10.566635; this version posted November 10, 2023. The copyright holder for this preprint (which was not certified by peer review) is the author/funder, who has granted bioRxiv a license to display the preprint in perpetuity. It is made available under aCC-BY 4.0 International license.

Supplementary Information for

FBXO24 ensures male fertility by preventing abnormal accumulation of membraneless granules in sperm flagella

Yuki Kaneda[1,2], Haruhiko Miyata[1,*], Zoulan Xu[1,2], Keisuke Shimada[1], Maki Kamoshita[1], Tatsuya Nakagawa[1,2], Chihiro Emori[1], Masahito Ikawa[1,2,3,4,5,*]

[1]Research Institute for Microbial Diseases, Osaka University, 3-1 Yamadaoka, Suita, Osaka 5650871 JAPAN.
[2]Graduate School of Pharmaceutical Sciences, Osaka University, 1-6 Yamadaoka, Suita, Osaka 5650871 JAPAN.
[3]The Institute of Medical Science, The University of Tokyo, 4-6-1 Shirokanedai, Minato-ku, Tokyo 1088639 JAPAN.
[4]Center for Infectious Disease Education and Research (CiDER), Osaka University, 2-8 Yamadaoka, Suita, Osaka 5650871 JAPAN.
[5]Center for Advanced Modalities and DDS (CAMaD), Osaka University, 2-8 Yamadaoka, Suita, Osaka 5650871 JAPAN.
*Corresponding authors: Haruhiko Miyata and Masahito Ikawa

Contact info
Email: hmiya003@biken.osaka-u.ac.jp (HM); ikawa@biken.osaka-u.ac.jp (MI)

bioRxiv preprint doi: https://doi.org/10.1101/2023.11.10.566635; this version posted November 10, 2023. The copyright holder for this preprint (which was not certified by peer review) is the author/funder, who has granted bioRxiv a license to display the preprint in perpetuity. It is made available under aCC-BY 4.0 International license.

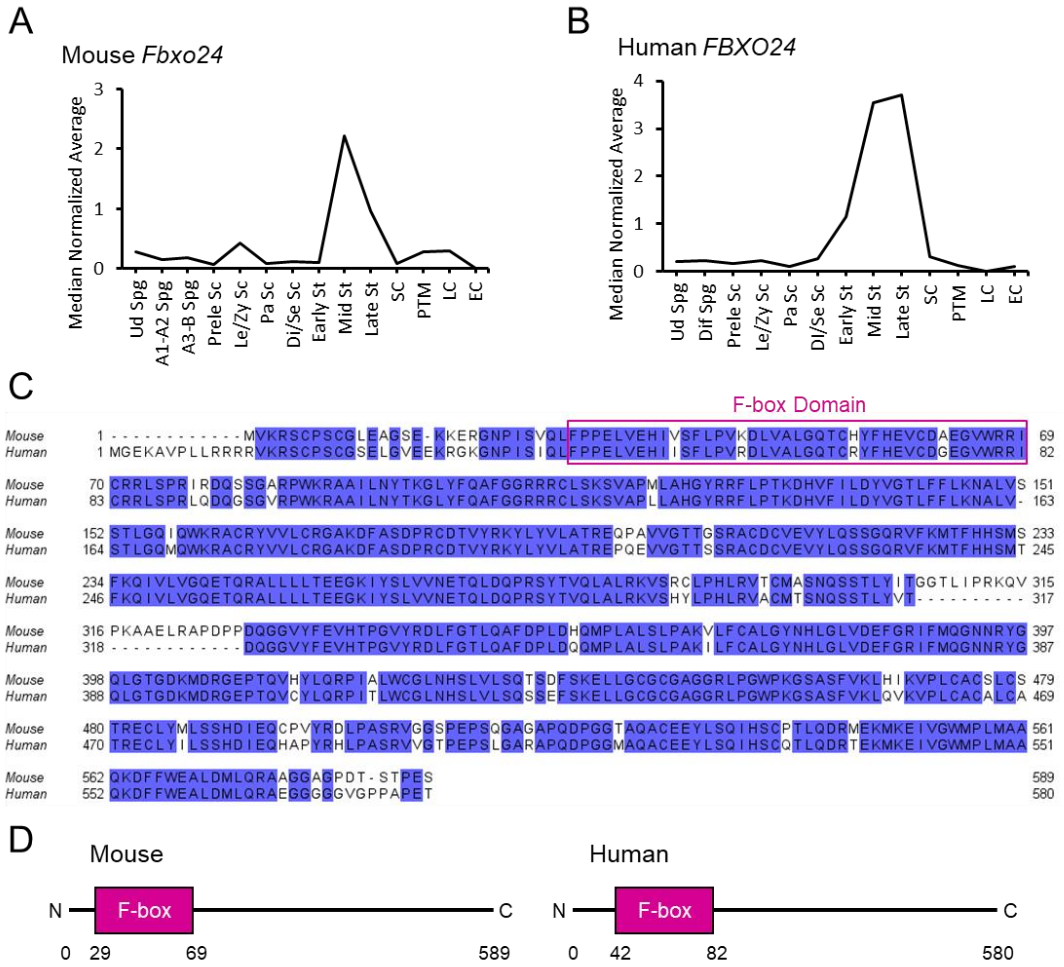

Figure S1. Characterization of mouse and human FBXO24. (*A*) Expression profile of mouse *Fbxo24* in spermatogenic cells is shown. Expression profiling was obtained from the testis single cell RNA-seq data set (Hermann et al., 2018). Ud spg: Undifferentiated spermatogonia, A1- A2 Spg: A1 and A2 differentiating spermatogonia, A3-B Spg: A3, A4, In, and B differentiating spermatogonia, Prele Sc: preleptotene spermatocytes, Le/Zy Sc: leptotene/zygotene spermatocytes, Pa Sc: pachytene spermatocytes, Di/Se Sc: diplotene/secondary spermatocytes, Early St: early round spermatids, Mid St: mid round spermatids, Late St: late round spermatids, SC: Sertoli cells, PTM: peritubular myoid cells, LC: Leydig cells, and EC: Endothelial cells. (*B*) Expression of human *FBXO24* in spermatogenic cells is shown. Expression profiling was obtained from the testis single cell RNA-seq data set (Hermann et al., 2018). Dif Spg: differentiating spermatogonia. (*C*) Pairwise sequence alignment of amino acids between mouse and human FBXO24. Dark blue highlight indicates identical amino acids. (*D*) The F-box domain of mouse and human FBXO24 was identified with a protein database search (SMART; http://smart.embl-heidelberg.de/).

bioRxiv preprint doi: https://doi.org/10.1101/2023.11.10.566635; this version posted November 10, 2023. The copyright holder for this preprint (which was not certified by peer review) is the author/funder, who has granted bioRxiv a license to display the preprint in perpetuity. It is made available under aCC-BY 4.0 International license.

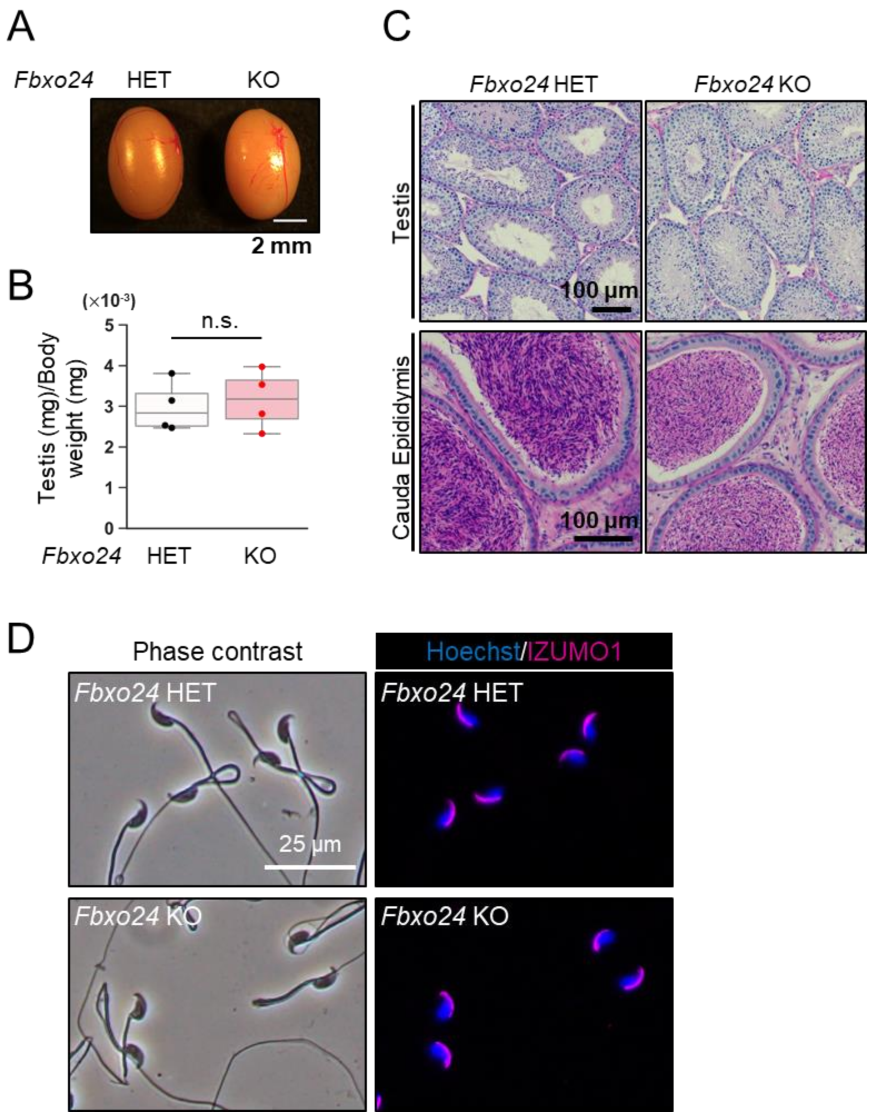

Figure S2. Histological analyses of *Fbxo24* KO testis and epididymis. (*A*) Gross morphology of testes obtained from *Fbxo24* heterozygous and KO males. (*B*) Test weight (mg)/body weight (mg) of *Fbxo24* heterozygous and KO mice. (*C*) PAS-hematoxylin staining of testes and cauda epididymis sections. (*D*) Spermatozoa obtained from the cauda epididymis were stained for IZUMO1 (magenta). Nuclei were stained with Hoechst 33342 (blue).

bioRxiv preprint doi: https://doi.org/10.1101/2023.11.10.566635; this version posted November 10, 2023. The copyright holder for this preprint (which was not certified by peer review) is the author/funder, who has granted bioRxiv a license to display the preprint in perpetuity. It is made available under aCC-BY 4.0 International license.

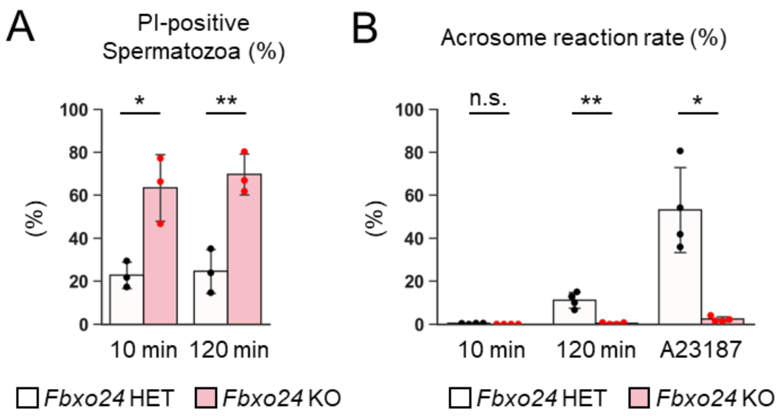

Figure S3. Lack of *Fbxo24* impairs sperm viability and the acrosome reaction. (*A*) Viability of cauda epididymal spermatozoa was assessed with propidium iodide (PI) at 10 min and 120 min incubation in TYH medium. (*B*) The acrosome reaction rates of cauda epididymal spermatozoa were assessed using RBGS Tg mice after 10 min and 120 min of incubation in TYH medium. To induce the acrosome reaction, Ca^{2+} ionophore A23187 was added to the medium after 120 min of incubation.

bioRxiv preprint doi: https://doi.org/10.1101/2023.11.10.566635; this version posted November 10, 2023. The copyright holder for this preprint (which was not certified by peer review) is the author/funder, who has granted bioRxiv a license to display the preprint in perpetuity. It is made available under aCC-BY 4.0 International license.

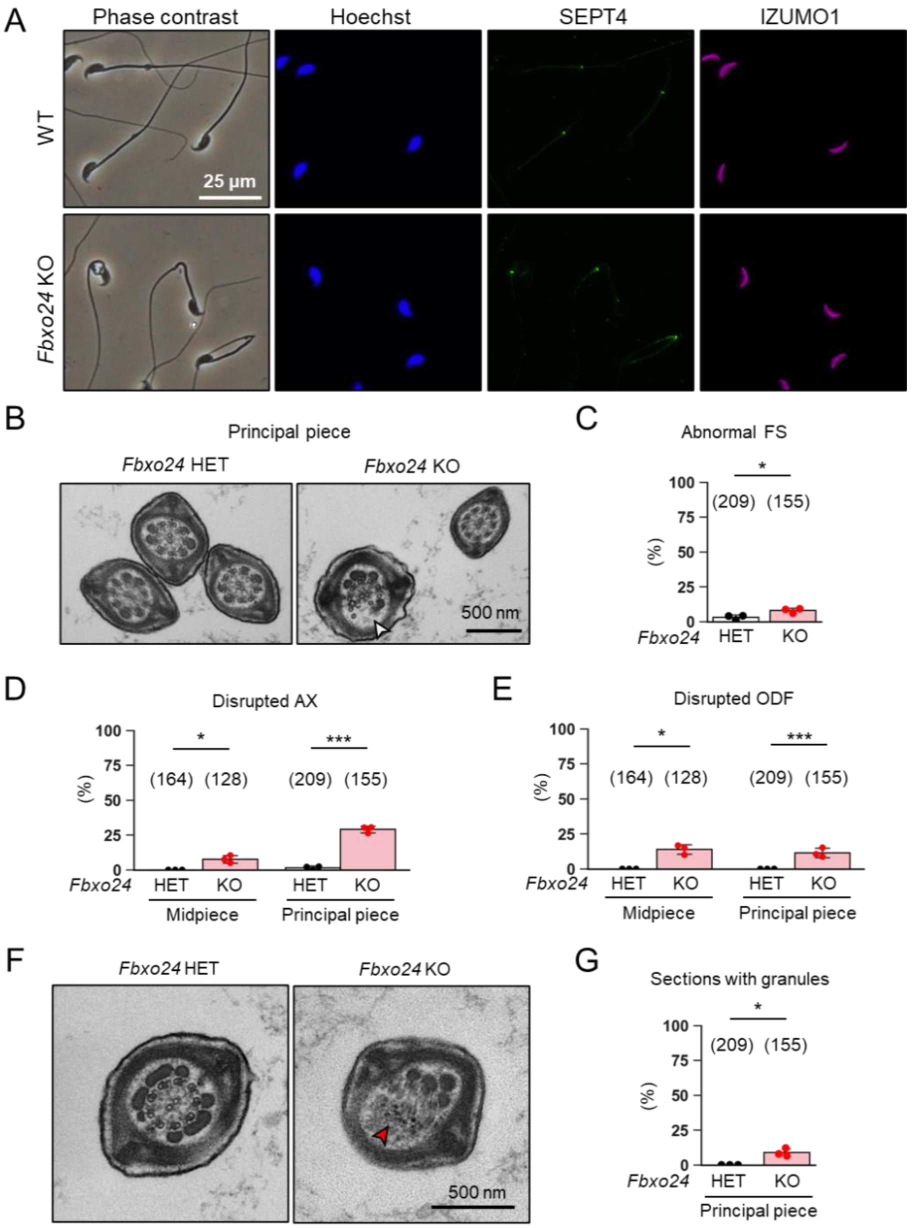

Figure S4. Sperm flagellum ultrastructure was disorganized in *Fbxo24* KO mice. (*A*) Localization of the annulus in cauda epididymal spermatozoa. Anti-SEPT4 antibody was used to visualize the annulus (green). Nuclei were detected with Hoechst 33342 (blue). The acrosome was detected with anti-IZUMO1 antibody (magenta). (*B*) Transmission electron microscopy (TEM) observation of the principal piece. A white arrowhead indicates a disruption of the axoneme and outer dense fiber (ODF). (*C*) Percentages of morphologically abnormal fibrous sheaths (FS) observed with TEM. The number of flagellar sections analyzed is shown above each bar. (*D*) Percentages of morphologically abnormal axonemes (AX) observed with TEM. The number of flagellar sections analyzed are shown above each bar. (*E*) Percentages of morphologically abnormal ODF observed with TEM. The number of flagellar sections analyzed are shown above each bar. (*F*) TEM observation of the principal piece. A red arrowhead indicates electron-dense granules. (*G*) Percentages of electron-dense granules observed in principal piece cross sections. The number of flagellar sections analyzed are shown above each bar.

bioRxiv preprint doi: https://doi.org/10.1101/2023.11.10.566635; this version posted November 10, 2023. The copyright holder for this preprint (which was not certified by peer review) is the author/funder, who has granted bioRxiv a license to display the preprint in perpetuity. It is made available under aCC-BY 4.0 International license.

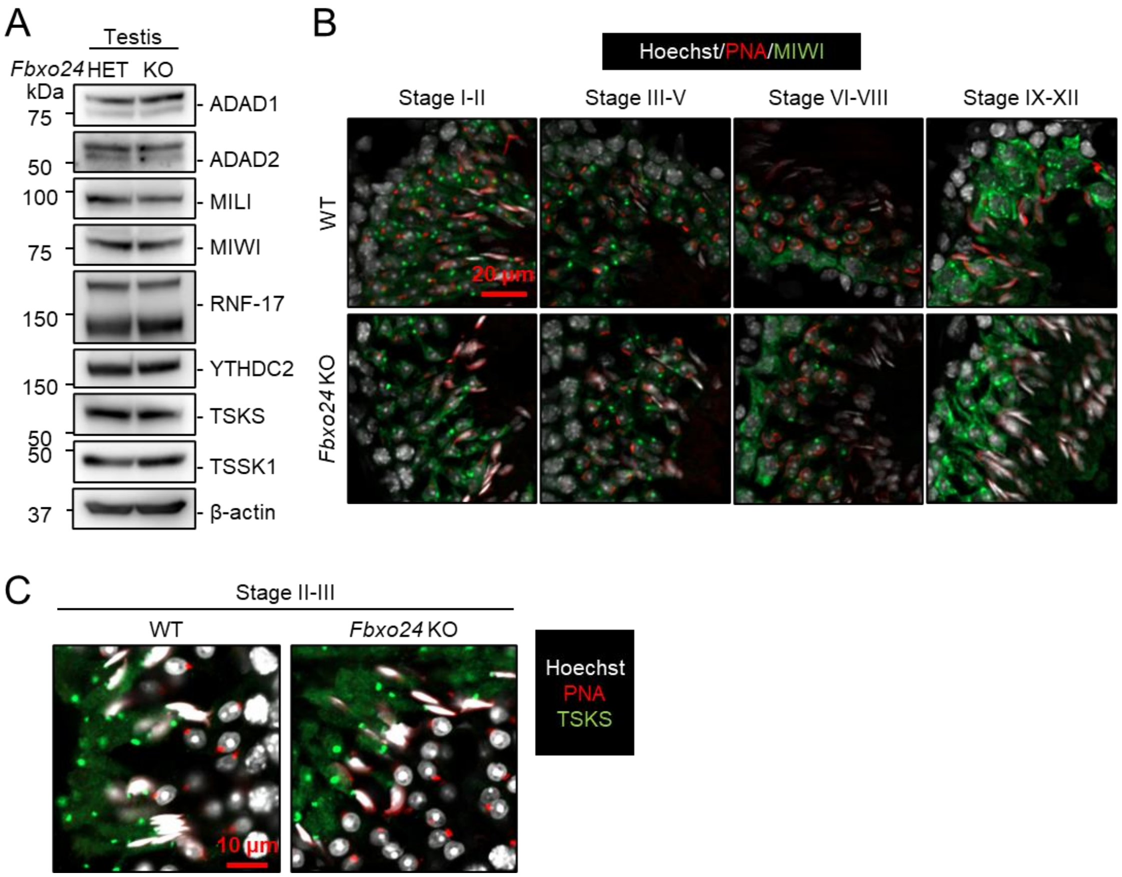

Figure S5. No clear differences were found in the amount and localization of RNP granule-related proteins between control and *Fbxo24* KO testes.
(*A*) Immunodetection of ADAD1, ADAD2, MILI, MIWI, RNF-17, YTHDC2, TSKS and TSSK1 in *Fbxo24* heterozygous and KO testes. β-actin was used as loading control. (*B*) Immunofluorescence observation of MIWI (green) in WT and *Fbxo24* KO testes. Acrosomes were stained with PNA (red) and nuclei were stained with Hoechst 33342 (white). (*C*) Immunofluorescence observation of TSKS (green) in WT and *Fbxo24* KO testes. Acrosomes were stained with PNA (red) and nuclei were stained with Hoechst 33342 (white).

bioRxiv preprint doi: https://doi.org/10.1101/2023.11.10.566635; this version posted November 10, 2023. The copyright holder for this preprint (which was not certified by peer review) is the author/funder, who has granted bioRxiv a license to display the preprint in perpetuity. It is made available under aCC-BY 4.0 International license.

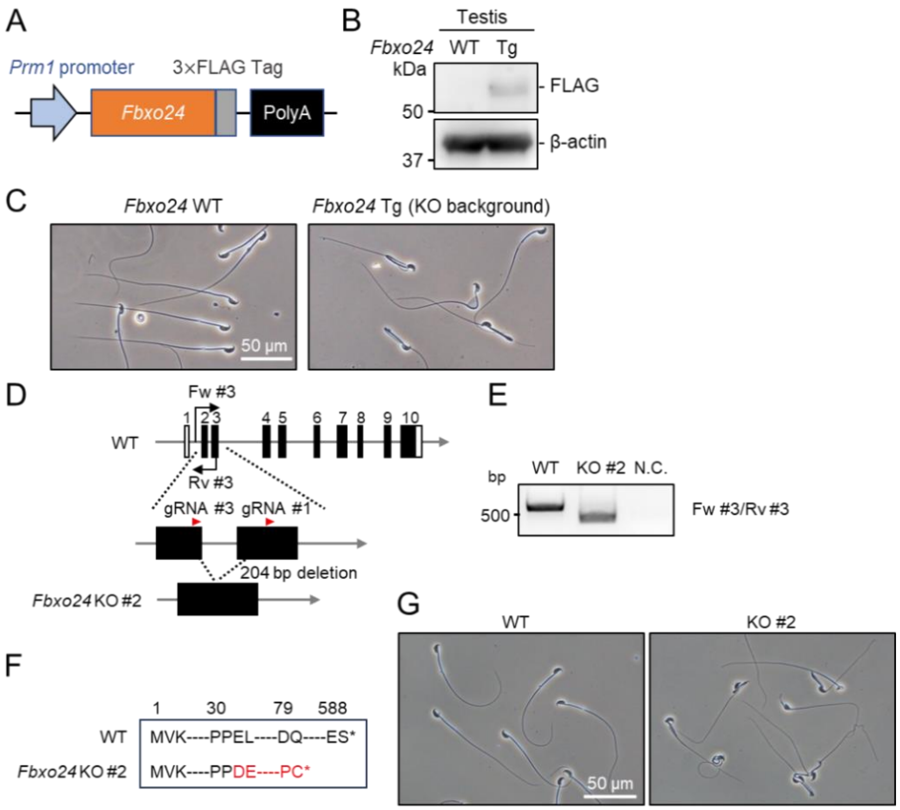

H

Identified Proteins	Quantitative Value (Normalized Total Spectra) Tg	WT
FBXO24	94	0
IPO5	68	0
STIP1	54	0
HSPA1B	41	0
HSPA4	36	0
SUGT1	34	0
DNAJC7	28	0
FKBP4	27	0
TUBB3	25	0
RPAP3	25	0
RUVBL2	24	0
SKP1	21	0
RUBCL1	21	0
DNAJA4	20	0
NUDCD3	20	0

Figure S6. Generation and analyses of *Fbxo24-3×FLAG* Tg mice. (*A*) Schematic of the *Fbxo24-3×FLAG* construct used to generate Tg mice. (*B*) Immunodetection of FBXO24-3×FLAG in the testis from the Tg mice. β-actin was used as a loading control. (*C*) Morphology of cauda epididymal spermatozoa. (*D*) Schematic for generating *Fbxo24* KO #2 mice using the CRISPR/Cas9 system. White boxes indicate untranslated regions while black boxes indicate protein coding regions. The gRNAs used are shown. Fw and Rv indicate the forward and reverse primer used for genotyping, respectively. (*E*) Genotyping of obtained *Fbxo24* mutant mice. Fw #3-Rv #3 primers in Fig. S6D were used. N.C. indicates negative control (water). *(F)* Predicted FBXO24 amino acid sequences of KO #2 mutant mice. The 204 bp deletion resulted in E32D mutation with a premature stop codon introduced 49 amino acids later. (G) Morphology of mature spermatozoa obtained from cauda epididymis. (*H*) The list of identified proteins by IP-MS analysis. The top 15 identified proteins in Tg are shown. The Quantitative Value (Normalized Total Spectra) was calculated using Scaffold proteome software. IgG-related proteins, which is likely from the antibody used for IP, were removed from the list.

bioRxiv preprint doi: https://doi.org/10.1101/2023.11.10.566635; this version posted November 10, 2023. The copyright holder for this preprint (which was not certified by peer review) is the author/funder, who has granted bioRxiv a license to display the preprint in perpetuity. It is made available under aCC-BY 4.0 International license.

Table S1. Primers and gRNAs used in this study.

primer	sequence (5'-3')
Fbxo24 Fw #1	tcctctggcaggtgaagcgc
Fbxo24 Fw #2	gatgtgttgattgtatgagacttcc
Fbxo24 Fw #3	actagcagcaagaagtccagg
Fbxo24 Rv #1	tctcgctcatgatagtcagg
Fbxo24 Rv #2	gaagtatggcaaagtcactgagtgc
Fbxo24 Rv #3	aagaatggcagctctcttcc
Fbxo24 Tg Fw	tgaagtgtgtgatgctgagg
Fbxo24 Tg Rv	gttcccgagtagccaaaaca
Fbxo24 Cloning Fw	atggatccgccgccatggtgaagcgcagctgcccttc
Fbxo24 Cloning Rv	gcgaattcgctctcgggagtcgaggtgtctgg
Actb Fw	catccgtaaagacctctatgccaac
Actb Rv	atggagccaccgatccaca

gRNA	target sequence (5'-3')
Fbxo24 gRNA #1	tgtggaggcgcatctgtcga
Fbxo24 gRNA #2	tcctgaaggaagtcgagccg
Fbxo24 gRNA #3	tcagttgttccccccagagc

bioRxiv preprint doi: https://doi.org/10.1101/2023.11.10.566635; this version posted November 10, 2023. The copyright holder for this preprint (which was not certified by peer review) is the author/funder, who has granted bioRxiv a license to display the preprint in perpetuity. It is made available under aCC-BY 4.0 International license.

Table S2. Antibodies used in this study.

antibodies	source	identifier
Rabbit anti-KPNA2	Abcam	ab84440
Rabbit anti-β-actin	MBL	PM053
Rabbit anti-human septin4	IBL	#18987
Rabbit anti-IPO5 (H-300)	SantaCruz	sc-11369
Rabbit anti-Miwi (G82)	Cell Sinaling Technology	#2079
Rabbit anti-ADAD1	(Lu et al., 2023)	
Rabbit anti-TSKS	(Shimada et al., 2023)	
Rabbit anti-TSSK1	(Shimada et al., 2023)	
Rat anti-ADAD2	(Lu et al., 2023)	
Rat anti-IZUMO1 monoclonal antibody	(Ikawa et al., 2011)	
Mouse anti-1D4 tag	A kind gift from Dr. Martin M. Matzuk	
Mouse anti-FLAG tag (M2)	Sigma-Aldrich	F1804
Mouse anti-α-tubulin (B-5-1-2)	Sigma-Aldrich	T5168
Mouse anti-acetylated tubulin	Sigma-Aldrich	T7451
Mouse anti-ADAM3 (F-4)	SantaCruz	sc-365288
Mouse anti-IPO5 (B-7)	SantaCruz	sc-55527
Mouse anti-β-actin (AC-15)	Abcam	ab6276
Mouse anti-KPNB1	Abcam	ab2811
Goat anti-rabbit IgG-HRP	Jackson ImmunoResearch	#111-036-045
Goat anti-rat IgG-HRP	Jackson ImmunoResearch	#112-035-167
Goat anti-mouse IgG-HRP	Jackson ImmunoResearch	#115-036-062
Goat anti-rabbit IgG-Alexa Fluor 488	Thermo Fisher Scientific	Cat# A11070
Goat anti-rabbit IgG-Alexa Fluor 546	Thermo Fisher Scientific	Cat# A11071
Goat anti-rat IgG-Alexa Fluor 488	Thermo Fisher Scientific	Cat# A11006
Goat anti-rat IgG-Alexa Fluor 546	Thermo Fisher Scientific	Cat# A11081
Goat anti-mouse IgG-Alexa Fluor 546	Thermo Fisher Scientific	Cat# A11017
Goat anti-mouse IgG-Alexa Fluor 546	Thermo Fisher Scientific	Cat# A11018

bioRxiv preprint doi: https://doi.org/10.1101/2023.11.10.566635; this version posted November 10, 2023. The copyright holder for this preprint (which was not certified by peer review) is the author/funder, who has granted bioRxiv a license to display the preprint in perpetuity. It is made available under aCC-BY 4.0 International license.

Legends for Movies S1 and S2

Movie S1 (separate file). Sperm motion of *Fbxo24* heterozygous mice was recorded after 120 min of incubation in TYH medium.

Movie S2 (separate file). Sperm motion of *Fbxo24* KO mice was recorded after 120 min of incubation in TYH medium.

SI References

Hermann BP, Cheng K, Singh A, Roa-De La Cruz L, Mutoji KN, Chen IC, Gildersleeve H, Lehle JD, Mayo M, Westernströer B, Law NC, Oatley MJ, Velte EK, Niedenberger BA, Fritze D, Silber S, Geyer CB, Oatley JM, McCarrey JR. 2018. The Mammalian Spermatogenesis Single-Cell Transcriptome, from Spermatogonial Stem Cells to Spermatids. *Cell Rep* **25**:1650-1667.e8. doi:10.1016/j.celrep.2018.10.026

Ikawa M, Tokuhiro K, Yamaguchi R, Benham AM, Tamura T, Wada I, Satouh Y, Inoue N, Okabe M. 2011. Calsperin is a testis-specific chaperone required for sperm fertility. *J Biol Chem* **286**:5639–5646. doi:10.1074/jbc.M110.140152

Lu Y, Nagamori I, Kobayashi H, Kojima-Kita K, Shirane K, Chang H, Nishimura T, Koyano T, Yu Z, Castañeda JM, Matsuyama M, Kuramochi-Miyagawa S, Matzuk MM, Ikawa M. 2023. ADAD2 functions in spermiogenesis and piRNA biogenesis in mice. *Andrology* **11**:698–709. doi:10.1111/andr.13400

Shimada K, Park S, Oura S, Noda T, Morohoshi A, Matzuk MM, Ikawa M. 2023. TSKS localizes to nuage in spermatids and regulates cytoplasmic elimination during spermiation. *Proc Natl Acad Sci U S A* **120**:e2221762120. doi:10.1073/pnas.2221762120
